# Variability in Practice of Buprenorphine Treatment by Emergency Department Operational Characteristics

**DOI:** 10.5811/westjem.18019

**Published:** 2024-06-11

**Authors:** Grant Comstock, Natalia Truszczynski, Sean S. Michael, Jason Hoppe

**Affiliations:** * Medical College of Wisconsin, Department of Emergency Medicine, Division of Medical Toxicology, Milwaukee, Wisconsin; † University of Colorado School of Medicine, Aurora, Colorado; ‡ University of Colorado School of Medicine, Department of Emergency Medicine, Aurora, Colorado; § University of Colorado School of Medicine, Department of Emergency Medicine, Division of Medical Toxicology and Pharmacology, Aurora, Colorado

## Abstract

**Introduction:**

We sought to describe emergency department (ED) buprenorphine treatment variability among EDs with varying operational characteristics.

**Methods:**

We performed a retrospective cohort study of adult patients with opioid use disorder discharged from 12 hospital-based EDs within a large healthcare system as a secondary data analysis of a quality improvement study. Primary outcome of interest was buprenorphine treatment rate. We described treatment rates between EDs, categorized by tertile of operational characteristics including annual census, hospital and intensive care unit (ICU) admission rates, ED length of stay (LOS), and boarding time. Secondary outcomes were ED LOS and 30-day return rates.

**Results:**

There were 7,469 unique ED encounters for patients with opioid use disorder between January 2020–May 2021, of whom 759 (10.2%) were treated with buprenorphine. Buprenorphine treatment rates were higher in larger EDs and those with higher hospital and ICU admission rates. Emergency department LOS and 30-day ED return rate did not have consistent associations with buprenorphine treatment.

**Conclusion:**

Rates of treatment with ED buprenorphine vary according to the operational characteristics of department. We did not observe a consistent negative relationship between buprenorphine treatment and operational metrics, as many feared. Additional funding and targeted resource allocation should be prioritized by departmental leaders to improve access to this evidence-based and life-saving intervention.

Population Health Research Capsule What do we already know about this issue?

*Understanding the impact of emergency department (ED) buprenorphine on operations can inform resource allocation decisions for ED buprenorphine program development.*

What was the research question?

*How does ED buprenorphine impact operations? How do ED operational characteristics impact treatment rates?*

What was the major finding of the study?

*A small number of patients with opiod use disorder were prescribed buprenorphine (2.5% in small hospitals, 11.6% in large hospitals). ED length of stay and 30-day return did not differ based on buprenorphine treatment.*

How does this improve population health?

*Departmental leadership can prioritize ED buprenorphine program development without fear of negative operational impact to increase access to life saving treatment.*

## INTRODUCTION

The opioid crisis is a worsening public health emergency, with over 80,000 opioid-involved overdose deaths in the US in 2021, and it is unlikely to abate in the absence of effectively implemented harm reduction and treatment strategies.^1^ Buprenorphine is an effective, evidence-based treatment resulting in increased abstinence from illicit opioid use and decreased opioid-related mortality.^2^^,^^3^ Emergency department (ED) buprenorphine treatment is an evidence-based practice and has been associated with increased follow-up and reduced illicit drug use and medical costs.^4^^,^^5^Although buprenorphine prescribing from EDs has increased in recent years, prescribing still lags far behind the apparent need, with disparities by payer status, race, and ethnicity.^6^^,^^7^

Improved implementation relies on identification and removal of barriers, providing resources for patients and clinicians, and dispelling stigma and misperceptions.^8^ Emergency department operational considerations, including perceptions of insufficient time and increased ED return visits, are commonly cited as perceived barriers to implementation.^9^ However, the real-world interplay between ED buprenorphine initiation and ED operations is not well described. Understanding the impact of ED buprenorphine treatment on ED clinical operational outcomes can inform decisions on resource allocation for ED buprenorphine program development. Conversely, barriers to implementation likely vary depending on the baseline operational performance of the department. Identification of operational characteristics of EDs with lower buprenorphine treatment rates would allow for targeted interventions.

We sought to describe the knowledge gap regarding ED buprenorphine treatment variability and operational barriers to implementation by 1) quantifying treatment rates between hospital EDs with different baseline operational characteristics, and 2) measuring the impact of ED buprenorphine treatment on operational metrics.

## METHODS

We performed a retrospective cohort study of adult (age ≥18) ED patients with opioid use disorder (OUD) discharged from any of the academic (one) or community (11) hospital-based EDs within a large healthcare system between January 2020–May 2021. The study was approved by our institutional review board for secondary data analysis of a completed quality improvement project.

To identify ED patients with OUD who may benefit from buprenorphine treatment, we applied an electronic health record (EHR) computable phenotype previously developed and validated by Chartash et al.^10^ Data were extracted by querying an ED analytics data mart populated by a nightly extract from the Epic Clarity (Epic Systems Corporation, Verona, WI) database. Patients were identified by searching from phenotype-specific diagnosis codes and ED chief complaints. Pertinent codes included International Classification of Diseases, 10th Rev, Clinical Modification (ICD-10) diagnostic codes relating to opioid use (T40.0*, T40.1*, T40.2*, T40.3*, T40.4*, T40.6*, and F11*) coded by either the treating clinician or subsequently by a medical coder. We additionally included patients not identified by ICD-10 diagnostic code ED chief complaints relating to opioid use. Chief complaint data is entered into the EHR at time of ED encounter from a prepopulated list, limiting our selection of search terms. Within the limits of our database, inclusion of encounters containing “opioid” or “naloxone” most closely reflected original phenotype terminology. Per phenotype, patients with the terms “benzodiazepine” or “alcohol” in their ED discharge diagnosis were excluded to limit false positive inclusion.

Encounter-level data extracted included the following: patient demographics; chief complaint; disposition; ED length of stay (LOS); doses of medications administered and prescribed; and follow-up information, including 30-day ED return rate and number of days until ED return within the same health system. All data was deidentified for analysis by the research team.

The primary outcome of interest was ED buprenorphine treatment, defined as percentage of patients administered buprenorphine during and/or prescribed buprenorphine as part of the ED visit among all patients with OUD identified by the EHR phenotype. After consulting with key administrative leaders and system stakeholders, we partitioned EDs based on operational characteristics including annual ED census; hospital and intensive care unit (ICU) admission rates; median ED LOS (time from ED arrival to ED departure); and median boarding time (time from admission order placed to ED departure). Hospitals were divided into tertiles for each characteristic. As no power or sensitivity analyses were performed, and our goal was descriptive and hypothesis-generating, we did not perform hypothesis-testing comparative analyses. Statistical analyses were performed using RStudio version 4.0.5 (RStudio PBC, Boston, MA) and IBM SPSS 26 (SPSS, Inc, Chicago, IL).

## RESULTS

The 2021 annual census for the 12 EDs ranged from 8,934 to 103,381 patients. Among 541,962 total unique ED encounters across sites from January 1, 2020–May 31, 2021, 7,469 (1.4%) visits were phenotype positive and constituted our study population, representing 5,637 unique patients, with a mean of 622 visits per ED site (range 51–2,547). Phenotype-positive patients were predominantly White (75.4%) and male (53.1%) (Table 1). A minority (759, 10.2%) were treated with buprenorphine during the ED encounter, 695 of whom (91.6%) received buprenorphine administered in the ED, 301 (40%) received a buprenorphine prescription, and 237 (31.2%) received both.

Buprenorphine was administered in the ED more frequently than it was prescribed at discharge, irrespective of operational characteristics. Larger hospitals and those with higher hospital and ICU admission rates had higher buprenorphine treatment rates (Table 2). EDs experiencing longer boarding times also trended toward higher rates of treatment.

**Table 1.** Characteristics of cohort of patients with opioid use disorder.

		ED buprenorphine treatment	
		Yes	No
Total encounters	541,962	759	6,710
Gender			
Male	243,961 (46.9)	436 (57.4)	3,528 (52.6)
Female	286,504 (52.9)	323 (42.6)	3,182 (47.4)
Not reporting	1,497 (0.3)	0	0
Race			
Black	55,975 (10.3)	91 (12)	610 (9.1)
White	374,736 (69.1)	537 (70.8)	5,094 (75.9)
Another race	111,251 (20.5)	131 (17.3)	1,006 (15)
Insurance status			
Self-pay	62,124 (11.5)	3 (0.4)	22 (0.3)
Medicare/Medicaid	307,513 (56.7)	589 (77.6)	4,955 (73.8)
Other insurer	163,489 (30.2)	162 (21.3)	1,648 (24.6)
VA	8,836 (1.6)	5 (0.7)	85 (1.3)
Average buprenorphine dose (mg)			
Administered	N/a	76.28	N/a
Prescribed	N/a	103.42	N/a
Encounters with naloxone prescription	N/a	268 (45.5)	1,041 (21)
*Percentages noted in parentheses

*ED*, emergency department; *VA*, Veterans Administration; *mg*, milligrams.

**Table 2.** Buprenorphine administration and prescription, categorized by emergency department operational characteristics.

	Average value per quantile (SD)	OUD visits (n = 7,469)	Buprenorphine administered (n = , %)	Buprenorphine prescribed (n = , %)	Administered and prescribed (n = , %)	Any buprenorphine (n = , %)	No buprenorphine (n = , %)
Annual ED census volume	Patients						
Small (n = 4)	11,424 (±2,413)	245	6 (2.4%)	1 (0.4%)	1 (0.4%)	6 (2.5%)	239 (97.6%)
Middle (n = 4)	29,351.5(±5,715)	1,245	61 (4.9%)	2 (0.2%)	2 (0.2%)	61 (4.9%)	1,184 (95.1%)
Large (n = 4)	69,739 (±30,656)	5,979	628 (10.5%)	298 (5%)	234 (3.9%)	692 (11.6%)	5,287 (88.4%)
ED number of beds	Beds						
Small (n = 4)	10.25 (±2.5)	245	6 (2.4%)	1 (0.4%)	1 (0.4%)	6 (2.5%)	239 (97.6%)
Middle (n = 4)	21 (±4.34)	1,245	61 (4.9%)	2 (0.2%)	2 (0.2%)	61 (4.9%)	1,184 (95.1%)
Large (n = 4)	49.5 (±17.23)	5,979	629 (10.5%)	298 (5%)	234 (3.9%)	692 (11.6%)	5,287 (88.4%)
Hospital admission rate	Rate						
Low (n = 4)	7.90% (±4.7%)	527	26 (4.9%)	1 (0.2%)	1 (0.2%)	26 (4.9%)	501 (95.1%)
Middle (n = 4)	16.98% (±1.8)	1,745	115 (6.6%)	6 (0.3%)	4 (0.2%)	117 (6.7%)	1,628 (93.3%)
High (n = 4)	27.41% (±3.2%)	5,197	554 (10.7%)	294 (5.7%)	232 (4.5%)	616 (11.9%)	4,581 (88.2%)
ICU admission rate	Rate						
Low (n = 4)	0.2% (±0.4%)	245	6 (2.5%)	1 (0.4%)	1 (0.4%)	6 (2.5%)	239 (97.6%)
Middle (n = 4)	1.8% (±0.3%)	2,027	135 (6.7%)	6 (0.3%)	4 (0.2%)	137 (6.8%)	1,890 (93.2%)
High (n = 4)	3.1% (±0.6%)	5,197	554 (10.7%)	294 (5.7%)	232 (4.5%)	616 (11.9%)	4,581 (88.2%)
ED length of stay	Minutes						
Short (n = 4)	106.3 (±8.6)	245	6 (2.5%)	1 (0.4%)	1 (0.4%)	6 (2.5%)	239 (97.6%)
Middle (n = 4)	149.8 (±4.7)	4,216	587 (13.9%)	287 (6.8%)	225 (5.3%)	649 (15.4%)	3,567 (84.6%)
Long (n = 4)	160.5 (±2.1)	3,008	102 (3.4%)	13 (0.4%)	11 (0.4%)	104 (3.5%)	2,904 (96.5%)
Median ED boarding time	Minutes						
Short (n = 4)	59.5 (±10.2)	245	6 (2.5%)	1 (0.4%)	1 (0.4%)	6 (2.5%)	239 (97.6%)
Middle (n = 4)	78.4 (±4.6)	1,437	91 (6.3%)	2 (0.1%)	2 (0.1%)	91 (6.3%)	1,346 (93.7%)
Long (n = 4)	110.5 (±24)	5,787	598 (10.3%)	298 (5.2%)	234 (4%)	662 (11.4%)	5,125 (88.6%)

*ED*, emergency department; *ICU*, intensive care unit; *OUD*, opioid use disorder.

Median ED LOS was similar among patients treated with buprenorphine versus not treated, although confidence intervals were wide (Table 3). Lower admission rate, smaller ED size, and smaller volume were associated with longer ED LOS for patients treated with buprenorphine. Proportion of patients returning to the ED within 30 days and time to ED return did not differ consistently based on treatment with buprenorphine.

**Table 3.** Emergency department operational outcomes by ED operational characteristics.

	ED OUD length of stay (minutes)			30-Day ED OUD return visits			Days before ED OUD return			
	Buprenorphine	95% CI	No buprenorphine	95% CI	Buprenorphine	No buprenorphine	Buprenorphine	95% CI	No buprenorphine	95% CI
Annual ED census volume										
Small (n = 4)	264	(148.2, 379.8)	181.6	(159.4, 203.8)	1 (0.1%)	81 (1.2%)	7	N/a	8.5	(6.8, 10.3)
Middle (n = 4)	250.4	(211.8, 289)	263.7	(251.1, 276.3)	14 (1.8%)	318 (4.7%)	8.7	(4.7, 12.8)	11.2	(10.2, 12.2)
Large (n = 4)	238	(216.9, 259.1)	275.6	(268.4, 282.7)	203 (26.8%)	1525 (22.7%)	11.5	(10.2, 12.67)	11	(10.6, 11.5)
ED number of beds										
Small (n = 4)	264	(148.2, 379.8)	181.6	(159.4, 203.8)	1 (0.1%)	81 (1.2%)	7	N/a	8.5	(6.8, 10.3)
Middle (n = 4)	250.4	(211.8, 289)	263.7	(251.1, 276.3)	14 (1.8%)	318 (4.7%)	8.7	(4.7, 12.8)	11.2	(10.2, 12.2)
Large (n = 4)	238	(216.9, 259.1)	275.6	(268.4, 282.7)	203 (26.8%)	1525 (22.7%)	11.5	(10.2, 12.7)	11	(10.6, 11.5)
Hospital admission rate										
Low (n = 4)	258	(212.4, 303.7)	245.2	(225.1, 265.3)	6 (0.8%)	156 (2.3%)	7.8	(4.7, 11)	10.4	(9.1, 11.8)
Middle (n = 4)	266	(224.9, 306.1)	287	(276.1, 297.9)	33 (4.4%)	461 (6.9%)	9.3	(6.3, 12.2)	10.2	(9.4, 10.9)
High (n = 4)	233.4	(210.7, 256.1)	266.8	(259.1, 274.6)	179 (23.6%)	1307 (19.5%)	11.7	(10.4, 13)	11.3	(10.8, 11.7)
ICU admission rate										
Low (n = 4)	264	(148.2, 379.8)	181.6	(159.4, 203.8)	1 (0.1%)	81 (1.2%)	7	N/a	8.5	(6.8, 10.3)
Middle (n = 4)	264.1	(228.8, 299.5)	289.2	(278.9, 299.6)	38 (5%)	536 (8%)	9.1	(6.5, 11.7)	10.5	(9.8, 11.2)
High (n = 4)	233.4	(210.7, 256.1)	266.8	(259.1, 274.6)	179 (23.6%)	1307 (19.5%)	11.7	(10.4, 13)	11.3	(10.8, 11.7)
ED length of stay										
Short (n = 4)	264	(148.2, 379.8)	181.6	(159.4, 203.8)	1 (0.1%)	81 (1.2%)	7	N/a	8.5	(6.8, 10.3)
Middle (n = 4)	225.8	(205.2, 246.3)	279.7	(271.6, 287.9)	187 (24.6%)	1059 (15.8%)	11.3	(10.1, 12.6)	11.1	(10.6, 11.6)
Long (n = 4)	321.7	(261.8, 381.6)	265.5	(255.8, 275.3)	30 (4%)	784 (11.7%)	11	(7.9, 14.1)	11	(10.4, 11.6)
Median ED boarding time										
Short (n = 4)	264	(148.2, 379.8)	181.6	(159.4, 203.8)	1 (0.1%)	81 (1.2%)	7	N/a	8.5	(6.8, 10.3)
Middle (n = 4)	285.9	(242.6, 329.2)	300.6	(285.8, 315.4)	27 (3.6%)	370 (5.5%)	9	(6, 12)	11.4	(10.5, 12.3)
Long (n = 4)	232.6	(211, 254.1)	266.2	(259.3, 273.1)	190 (25%)	1473 (22%)	11.6	(10.3, 12.9)	11	(10.5, 11.4)

*ED*, emergency department; *ICU*, intensive care unit; *OUD*, opioid use disorder.

## DISCUSSION

Within this single health system, we observed that ED buprenorphine treatment rates varied according to the baseline operational characteristics of the ED, which may be a proxy for the progressiveness or philosophical approach of a given ED’s local champions and leadership team. We observed lower rates of buprenorphine treatment in EDs with smaller annual census and lower acuity (as measured by overall and ICU admission rates), which are presumably practice settings where there may be less perception of insufficient time. However, smaller EDs are less likely to have multiple prescribing clinicians working simultaneously. Prior studies have suggested that practice variation portends lower quality care and inequities in access to effective treatment for OUD.^11^^,^^12^ Our data supports the need for interventions designed to promote buprenorphine treatment in smaller, lower acuity EDs to narrow this variation.

Buprenorphine treatment did not appear to have a consistent association with ED LOS, in contrast to commonly cited barriers.^9^ Thirty-day return rates and time to ED return were similar between patients with OUD, regardless of their treatment with buprenorphine, a far cry from cited fears of EDs becoming “overrun” by patients seeking buprenorphine refills.^13^

Support from key departmental stakeholders is a repeatedly identified facilitator for implementing ED buprenorphine programs, and our observations corroborate this finding.^13^ If LOS and ED return rate are relatively unaffected by ED buprenorphine treatment, this has important implications that might allow departmental leaders to promote greater resourcing and mitigate some of their apprehensions to facilitate buprenorphine treatment without fear of negative operational impacts.

## LIMITATIONS

Our study intent was descriptive and should be considered hypothesis-generating. The use of secondary data limited our ability to power the study, and 95% confidence intervals were often wide. Treatment rates may be falsely lowered by the presence of patients already on treatment and, therefore, not offered ED-based buprenorphine, although this would be unlikely to impact comparison between sites. Our dataset is also limited by size and confinement to a single health system as well as lack of patient diversity, which may limit generalizability. Importantly, unmeasured operational and cultural factors may prompt any given ED’s leadership team to support buprenorphine treatment, and many of those same factors likely influence the general operational characteristics of the ED.

While this health system operates on a common EHR, clinicians are all employed by the health system, and incentives at all sites are tied to relative value units, there is a strong element of local control over the operations of each local ED, with little admixing of staff or operational processes between them. Nevertheless, clinicians may have moved between sites or worked at multiple sites. There may be unmeasured temporal trends during the study period, and a minority of more progressive EDs (including only one academic ED) may have contributed disproportionately to our findings. Finally, our partitioning of EDs by organizational metrics was based on internal comparisons specific to our healthcare system. Attempts to use national benchmarking data from the Academy of Administrators in Academic Emergency Medicine or Emergency Department Benchmarking Alliance were unsuccessful, as national mean and median metrics created severely uneven group sizes. While our approach may limit generalizability to other healthcare systems, it still may have implications for future hypothesis-testing research.

## CONCLUSION

The evidence supporting the societal benefit of ED initiation of buprenorphine for patients with opioid use disorder is clear, but ED operational leadership and stakeholder buy-in is key to increasing implementation. Based on our study results, we hypothesize that ED buprenorphine treatment rates varied based on operational characteristics of EDs, with lower treatment rates at smaller, lower acuity facilities. We did not observe consistent differences in length of stay or return visits. Future research will allow departmental leadership to continue prioritizing the evidence-based practice of ED buprenorphine treatment to decrease variability while improving quality of care and access to life-saving treatment for patients with OUD. This is particularly important given the recent removal of the X-waiver requirement.
